# Could differences in implicit attitudes to sexual concurrency play a role in generalized HIV epidemics?

**DOI:** 10.12688/f1000research.14951.2

**Published:** 2018-10-18

**Authors:** Chris R. Kenyon, Kenny Wolfs, Kara Osbak, Maleeto Malataliana, Guido Van Hal, Sizwe Zondo, Jacques van Lankveld

**Affiliations:** 1 HIV/STI Unit, Institute of Tropical Medicine, Antwerp, 2000, Belgium; 2 Faculty of Psychology and Educational Science, Open University of the Netherlands, Heerlen, The Netherlands; 3 Department of Psychology, Rhodes University, Grahamstown, South Africa; 4 Medical Sociology and Health Policy, University of Antwerp, Antwerp, Belgium

**Keywords:** concurrency, HIV, sexual networks, implicit association

## Abstract

**Background**: Sexual partner concurrency has been implicated in the genesis of generalized HIV epidemic in South Africa. Most South Africans, however, disapprove of concurrency in surveys. These surveys test individuals’ explicit attitudes which are susceptible to a number of important biases such as the social desirability bias. Assessment of implicit cognitions have been found to be better predictors of behaviour in socially sensitive domains. We hypothesized that South Africans may have implicit attitudes more tolerant of concurrency than lower concurrency prevalence populations.

**Methods:** To test this hypothesis, we developed a concurrency-implicit association test (C-IAT) and compared the C-IATs of samples of South African and Belgian university students.

**Results:** We found a large and statistically significant difference in the C-IAT between the South Africans (D600-score = -0.009, indicating absence of preference for concurrency or monogamy) and Belgians (D600-score = 0.783, indicating a strong preference for monogamy; t-test = 13.3; *P* < 0.0001). The effect size measure, Cohen’s d, was found to be 0.88, which is considered a large effect size in this field.

**Conclusions: **Our results are compatible with the thesis that differences in implicit attitudes to concurrency play a role in the genesis of generalised HIV epidemics.

## Introduction

A higher prevalence of sexual partner concurrency, were an individual has a series of overlapping sexual partners at once, is one of the factors implicated in the genesis of generalized HIV epidemics in Southern and Eastern Africa ^1– 3^. Qualitative research from the region has argued that a tolerance of concurrency plays an important role in generating high concurrency rates ^4– 9^. A quantitative analysis of South African survey data, however, found that most men and women disapproved of concurrency ^9^. This discrepancy may be partly explained by the way that the social desirability bias may affect the accuracy of self-reported data pertaining to socially sensitive topics such as sexual norms ^10– 14^. Respondents to surveys asking about attitudes to sexual partner concurrency may consider that the interviewer holds negative attitudes towards concurrency. They may therefore bias their reported attitudes towards concurrency towards that of the interviewer. Measures of implicit cognition assess cognitive processes less available to introspection and are thus less affected by these problems. Several studies have found implicit measures to be better predictors of behavior than explicit measures in these sensitive domains ^10, 13– 15^. In a previous study, we developed a concurrency implicit association test (C-IAT) and tested it on a sample of 869 Belgian students ^16^. The students revealed a strong implicit preference for monogamy as opposed to concurrency. No differences in C-IAT were found between men and women, but men who have sex with men and women who have sex with women were found to have a somewhat weaker implicit preference for monogamy than heterosexual men and women ^16^.

In this study, we compare the results from this Belgian study with those obtained from a similar sample of South African students. We assess: (i) if implicit and explicit norms towards concurrency differ between Belgian and South African university students, (ii) if the variation between these two populations involves a difference in behavior of ‘core-groups’ or general population shifts (iii) the correlation between implicit and explicit attitudes to concurrency and reporting that one has engaged in concurrency at both individual and population levels. Our rationale for exploring if the variation between these two populations involves a difference in behavior of ‘core-groups’ or general population shifts is based on the work of Rose and others ^17– 19^. They argued that if one finds a bimodal distribution in behavior 'A' in population 'B' compared to a normal distribution in a comparison population 'C' then this finding would be compatible with the existence of a core-group with higher risk behaviour in population 'B' being responsible for some of the differences in behavior 'A' between the two groups. The concept of a 'core-group' is well established in the HIV field and typically refers to a subpopulation with a high level of sexual network connectivity (conferred by features such as partner concurrency and rate of partner change) that contributes disproportionately to the spread of HIV in that population ^17^.

## Methods

### Concurrency-IAT description

Implicit Association Tests (IATs) are reaction-time measures that tap implicit associations without requiring conscious introspection ^20^. We developed a Concurrency-IAT (C-IAT) that measures the implicit associations that individuals hold towards concurrency in relation to monogamy. Our C-IAT was constructed using the attribute categories “positive/negative” and the target categories “monogamy/multiple partners.” Participants had to categorize words as either positive or negative and pictures as either depicting two people in a monogamous relationship or two people of which one had another partner.

Our C-IAT consisted of five different blocks. The C-IAT was programmed in OpenSesame, an open source program for reaction time experiments, for the offline version that was used in English in South Africa ^21^. The online Dutch-language IAT used in in Belgium was hosted on the Project Implicit® Web site. The full C-IATs as well as all the words and images used in their construction can be obtained from Kenyon *et al*. ^16^

### Explicit questionnaire

After completing the IAT the students were asked to complete a questionnaire pertaining to their sexual behavior and explicit attitudes to concurrency. These questions (variables they are intended to define) included: *How many sex partners do you have?* (Point prevalence concurrency); *Where there any other times in your life when you had more than one sex partner at a time?* (Life time concurrency). Three questions investigating explicit attitudes towards concurrency were assessed using a scale from 1 (strongly disagree) to 5 (strongly agree): *It’s okay to have sex with others as long as your main partner does not find out?* (Concealed concurrency); *If you are in a sexual relationship with someone, it’s okay to have sex with others as long as you are honest with your main partner about this?* (Liberalist concurrency); *If my main partner has other sex partners, it is okay for me to have other partners as well?* (Reactive concurrency) ^22^. The questions used in this questionnaire are available from Kenyon *et al*. ^16^

### Procedure/protocol

All procedures were approved by the Institutional Review Board of the Institute of Tropical Medicine (Antwerp) and the Ethics Committees of the University of Antwerp and Rhodes University.

In both countries all students at the two participating Universities were eligible for study inclusion. In Belgium they were recruited via an email sent to the entire student body. This was not possible in South Africa and thus students were recruited via posters and word of mouth.

### South Africa

All participants were tested independently either in the Department of Psychology or in a secure and quiet room at the Rhodes University library. After they had signed the informed consent form, students were first asked to perform the C-IAT behind a computer in the above mentioned locations. After students completed the C-IAT, they proceeded to answer the explicit, paper-and-pencil questionnaire measures. 

### Belgium

The entire protocol was conducted online. Students received a link to the study website via the recruitment e-mail. The first step on the study website was signing the informed consent form. They then completed the C-IAT, and after this the explicit questionnaire.

For both student populations, the IAT and explicit measures took between 15 and 20 minutes to complete.

### Statistical analysis

D600-scores of the IAT were calculated according to the standard protocol suggested by Greenwald *et al*. ^23, 24^ Scores usually vary between -2 and +2, indicating strong implicit preferences for concurrency and monogamy, respectively, with zero indicating absence of preference. The minimum response time was 400 ms, the maximum response time was 2500 ms. Any responses below this interval were omitted while any responses above this interval were recoded to 2500 ms. Incorrect answers got a penalty of 600 ms.

We compare the distributions of C-IAT (D600-scores) scores between Belgium and South Africa visually using histograms and statistically using t-tests for independent samples. In keeping with standard practice in this field, we used Cohen’s d as a measure of effect size. Cohen’s d was calculated by dividing the South African minus the Belgian mean difference D600 by the pooled standard deviation. Pearson’s correlation was used to test the correlations between implicit and explicit attitudes as well as between these two and self-reported point-prevalence of concurrency. Chi-squared and t-tests were used to test differences between categorical and continuous variables. All analyses were repeated stratified by gender. There were differences by gender in self-reported concurrency and explicit (but not implicit) attitudes towards concurrency. These differences were, however, congruent between Belgium and South Africa and did not affect the results. As a result, only unstratified results are reported.

*Population level analyses*: Sexual norms and behaviors such as concurrency have been shown to vary between different sexual orientations ^17, 25– 27^. This provided the rationale for using Spearman’s correlation to assess the population level correlations between the point-prevalence of concurrency and intrinsic (mean D600) and extrinsic (mean values for each of the 3 variables considered separately) attitudes. The populations were defined according to self-reported sexuality by country and gender. Only populations with n > 10 were utilized for the analyses.

All analyses were performed in Stata 13 (StataCorp LP, College Station, TX, USA). 

## Results

A total of 869 students in Belgium and 70 in South Africa participated. The demographic characteristics of the populations are detailed in Table 1. The South African students reported more sexual partners in the past year than the Belgians (mean 3.5 and 1.4, respectively, *P* < 0.001), a higher point-prevalence of concurrency (38.7% and 3.0%, *P* < 0.001), ever having engaged in concurrency (61.5% and 22.2%, *P* < 0.001) and partner concurrency (50.8% and 16.9%, *P* < 0.001; Table 1).

**Table 1.** Characteristics of study participants No. (%)/Mean [Standard Deviation].

	Belgium	South Africa
***N***	869	70
**Sex**		
**Men**	310 (37.1)	30 (34.5)
**Women**	526 (62.9)	39 (65.5)
**Age - mean [SD]**	22.94 [5.22]	22.09 [2.54]
**Race/Ethnicity**		***
**African/Black**	6 (0.7)	49 (70)
**European/White**	819 (97.6)	10 (14.3)
**Asian**	8 (1.0)	3 (2.9)
**Other**	6 (0.7)	9 (12.9)
**Sexual Orientation**		***
**Heterosexual**	733 (87.4)	52 (74.3)
**WSW** ^#^	20 (2.4)	4 (5.7)
**MSM** ^#^	32 (3.8)	12 (17.1)
**Other**	54 (6.4)	4 (2.9)
**Sexual behaviour**		
***N* partners last year – mean [SD]**	1.40 [2.57]	3.5 [3.13] **
**Current concurrency**	25 (3.0)	24 (38.7) **
**Ever concurrency**	184 (22.2)	40 (61.5) **
**Partner concurrency**	139 (16.9)	33 (50.8) **
**Explicit norms**		
**Concealed concurrency [SD]**	1.39 [0.68]	2.04 [1.22] **
**Liberalist concurrency [SD]**	2.56 [1.27]	1.91 [1.09] **
**Reactive concurrency [SD]**	2.41 [1.23]	2.36 [1.26]
**Implicit norms**		
**D600 [SD]**	0.783 [0.406]	-0.009 [0.425] ***

* P < 0.05, ** P < 0.001, *** P < 0.0001 (P-values are for comparisons between South Africa and Belgium).

# WSW – Woman who has sex with women, MSM – Man who has sex with men

### IAT results

**Figure 1.**
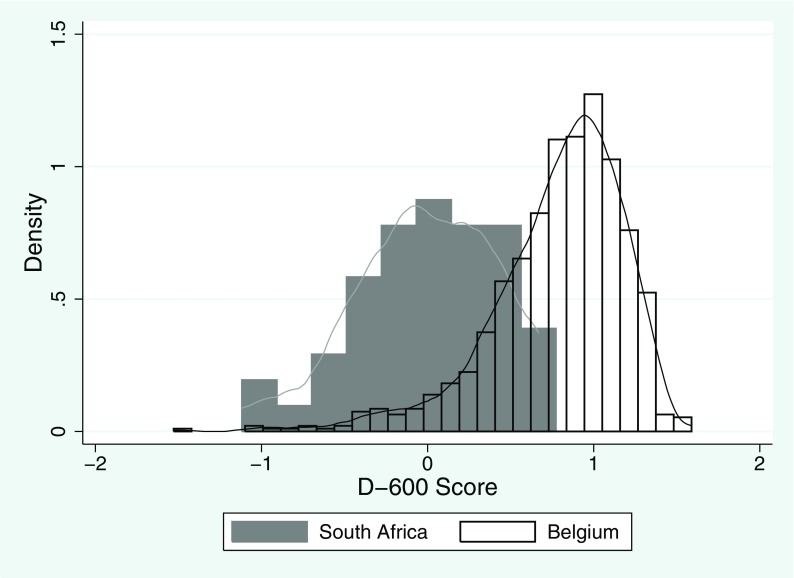
Distributions of D600 scores for South African and Belgian students (Positive and negative scores indicate preferences for monogamy and concurrency respectively).

The IAT results for the South African and Belgian populations both approximated normal distributions with similar standard deviations (SD) = 0.40 and 0.42 respectively; Figure 1). There was a large and statistically significant difference in the C-IAT between the South Africans (D600-score = -0.009, indicating absence of preference for concurrency or monogamy) and Belgians (D600-score = 0.783, indicating a strong preference for monogamy; t-test = 13.3; *P* < 0.0001). The effect size measure, Cohen’s d, was found to be 0.88 which is considered a large effect size in this field ^10^.

There was no difference in mean C-IAT score between men and women in Belgium (-0.81, SD = 0.39 and -0.78, SD = 0.40, respectively) or South Africa (0.05, SD = 0.46 and -0.01, SD = 0.40, respectively) 

### Explicit attitudes

The differences in implicit associations between countries were larger than those for explicit associations: South Africans were more pro-concealed-concurrency (d = 0.47), Belgians more pro-liberalist-concurrency (d = 0.27) and there was no difference in pro-reactive-concurrency (d = 0.03; Table 1).

### Association between self-reported point-prevalence concurrency and implicit and explicit attitudes

*Individual level*: Self-reported concurrency behavior was slightly more strongly associated with explicit (r = 0.08 to 0.58) than implicit (r = 0.05 to 0.11) attitudes to concurrency by country ( Table 2).

**Table 2.** Pearson's correlations between implicit and explicit attitudes towards concurrency.

	Belgium	South Africa
***N***	869	70
**Correlation point-concurrency vs IAT ^#^**	0.11 **	0.05
**Correlation point-concurrency vs explicit norms**		
**Concealed concurrency**	0.18 **	0.58 **
**Liberalist concurrency**	0.13 **	0.55 **
**Reactive concurrency**	0.08 *	0.49 **
**Correlation IAT ^#^ vs explicit norms**		
**Concealed concurrency**	0.17 **	0.18
**Liberalist concurrency**	0.20 **	0.22
**Reactive concurrency**	0.19 **	0.11

* P < 0.05, ** P < 0.001, *** P < 0.0001

# IAT - Implicit Association Tests

*Population level:* The prevalence of concurrency by sexual orientation was associated with the mean implicit attitude to concurrency (rho = 0.95, *P* = 0.0004, n = 8). The same relationship was present when the analysis was restricted to the Belgian students (rho = 0.87, *P* = 0.024, n = 6). The association between extrinsic attitudes and concurrency was not statistically significant (concealed concurrency: rho = 0.65, *P* = 0.06; liberal concurrency: rho = -0.50, *P* = 0.171; reactive concurrency: rho = -0.03, *P* = 0.932).

STable 1: Concurrency implicit association tests Click here for additional data file. Copyright: © 2018 Kenyon CR et al. 2018 Data associated with the article are available under the terms of the Creative Commons Zero "No rights reserved" data waiver (CC0 1.0 Public domain dedication).

## Discussion

The IAT results for the study populations in South Africa and Belgium were both normally distributed with a similar variance. Belgium’s population curve was however relatively right-shifted. There was no evidence of a ‘core high risk group’ with a distribution outside of the Gaussian distribution in either country. This variation of distributions between different populations via right or left shifting the mean value (but retaining the same variance) mimics the findings of Rose and others for a wide variety of physical and mental health attributes and behaviors, including number of sex partners ^17– 19^. Rose’s interpretation of this relationship was that populations do not tolerate ‘excessive’ variations in norms and behaviors and thus distributions of these characteristics move up and down as a whole ^19^.

Similarly, large differences in implicit cognition via shifting the mean value whilst retaining the same variance have been found between different groups in other studies ^12, 23, 28^. One 34 country study, for example, found large differences in mean implicit gender-science stereotype scores between countries ^28^. Of interest these mean IAT scores were found to be better predictors of national sex differences in math and science achievement than corresponding explicit attitudes. Culturally determined differences in implicit attitudes are thought to emerge during childhood or early adolescence as individuals participate in the custom complexes of their cultures ^11, 28, 29^. Historical and anthropological analyses suggest that the shift from polygamy to monogamy in Southern Africa over the last 150 years did not reduce the number of partners men had. Non-marital and non-main partnerships were however driven underground ^4, 6, 8, 30^. This created the norm which - although heavily contested - maintains that it is acceptable for men to have ‘kwapeni’s’ (secret lovers) as long as their main partner does not find out ^5– 7^. In our study, we found that 20.0% of South Africans versus 1.4% of Belgians agreed with this statement ( *P* < 0.001). Because this is a sensitive topic it is possible that the IAT is providing an alternative measure of the acceptance of concurrency.

If our IAT results from South Africa are indeed reflective of a broader acceptance of concurrency than a population such as Belgium and this acceptance is causally linked to higher concurrency rates then the distribution of implicit responses to concurrency amongst South African students suggests that population level interventions would be required to address this issue. Current efforts targeting concurrency are largely focused on higher risk individuals which are unlikely to result in a shift in the population distribution in implicit attitudes to concurrency ^5, 27, 31^. One approach may be to follow the Know Your Network concurrency reduction intervention which was shown to be feasible and acceptable in a rural Kenyan population ^32^.

A limitation with the line of reasoning outlined above is the low correlation found between self-reported concurrency and implicit attitudes to concurrency. This may be because the implicit attitudes play little or no role in driving high concurrency rates. Alternatively, the C-IAT may constitute an important marker of a population-wide greater tolerance of concurrency which broadly enables higher concurrency rates but that other specific risk factors then determine which individuals will engage in concurrency ^33^. We found some support for this latter interpretation in the form of a population level correlation between implicit attitudes towards concurrency and the practice thereof. What this suggests is that there is a broader acceptance of concurrency in South Africa at an implicit level. This might play a role in determining the higher prevalence of concurrency in South Africa. Other factors such as previous experience of partner concurrency may then determine which specific individuals engage in concurrency ^34, 35^. Further study limitations include: a small sample size in South Africa; only samples from two countries were included in the study; and there were slight differences in how participants were recruited and tested. In South Africa, explicit questionnaires were completed on paper and pencil and the IAT was run offline. In Belgium, both the explicit questionnaire and IAT were offered online and could be completed from home. Respondents were self-selected and thus the samples cannot be regarded as representative of the entire university student or national populations. Finally, in the Belgian sample the nature of the web-based IAT meant that we could not exclude the possibility of multiple participations by respondents.

## Data availability

The data referenced by this article are under copyright with the following copyright statement: Copyright: © 2018 Kenyon CR et al.

Data associated with the article are available under the terms of the Creative Commons Zero "No rights reserved" data waiver (CC0 1.0 Public domain dedication). 

Dataset 1: STable 1: Concurrency implicit association tests 10.5256/f1000research.14951.d203426 ^36^

## Supplementary material

**Supplementary File 1- IAT Test**

Concurrency implicit association test.

**Supplementary File 2- Figures and words used in concurrency implicit association test.**
