# Supplementary material for: Could differences in implicit attitudes to sexual concurrency play a role in generalized HIV epidemics?
Source: F1000Res. 2018 Oct 18;7:608. Originally published 2018 May 17. [Version 2] doi: 10.12688/f1000research.14951.2 (PMC6221060; doi:10.12688/f1000research.14951.2)
Supplement: Supplementary file 3 [file f1000research-7-18230-s0002.tgz › beca560e-7f00-445c-97c8-7a10c1f00fd4_New_supplementary_S2.pptx]

## Slide 1
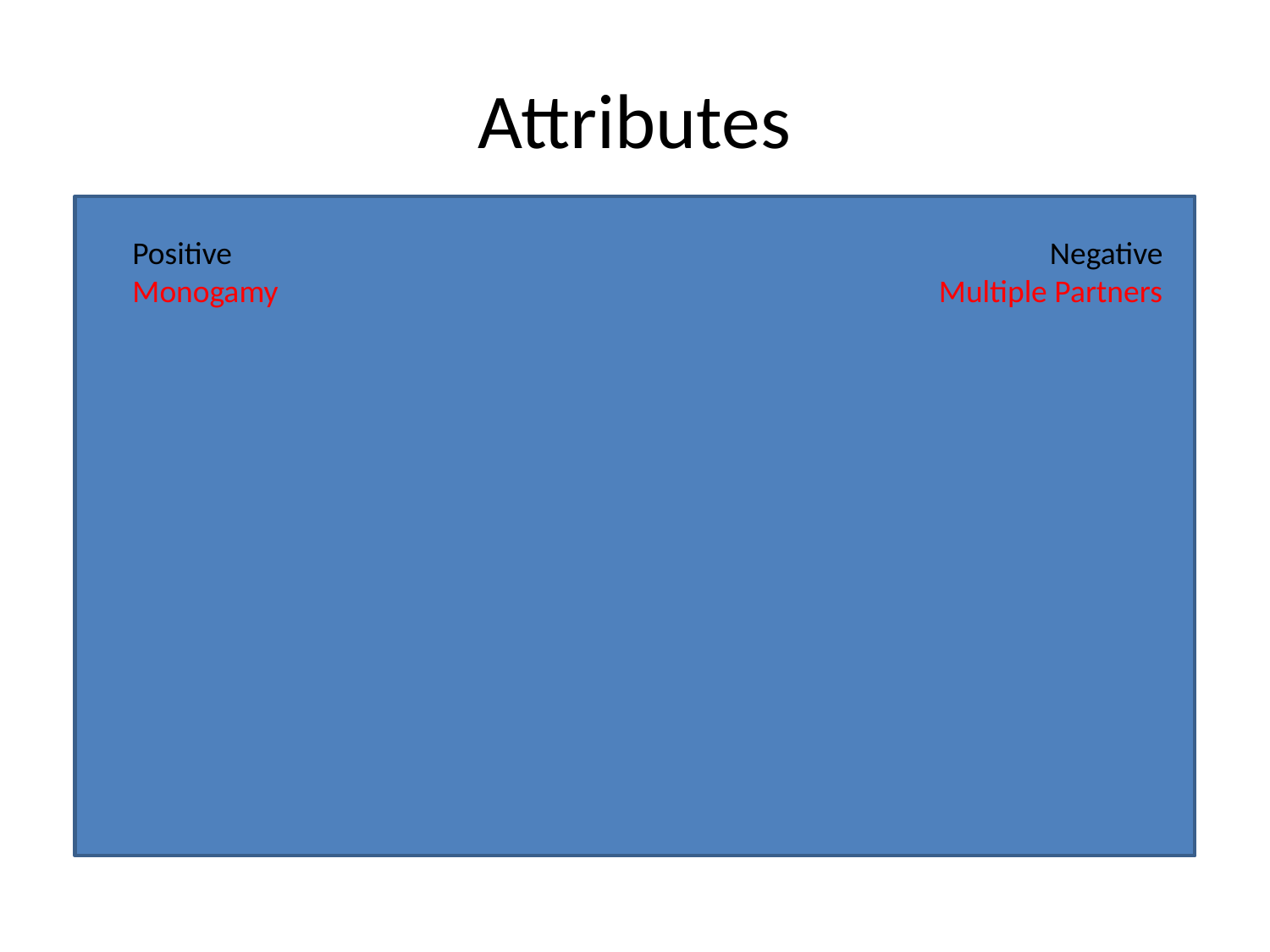

# Attributes
Positive
Monogamy
Negative
Multiple Partners

## Slide 2
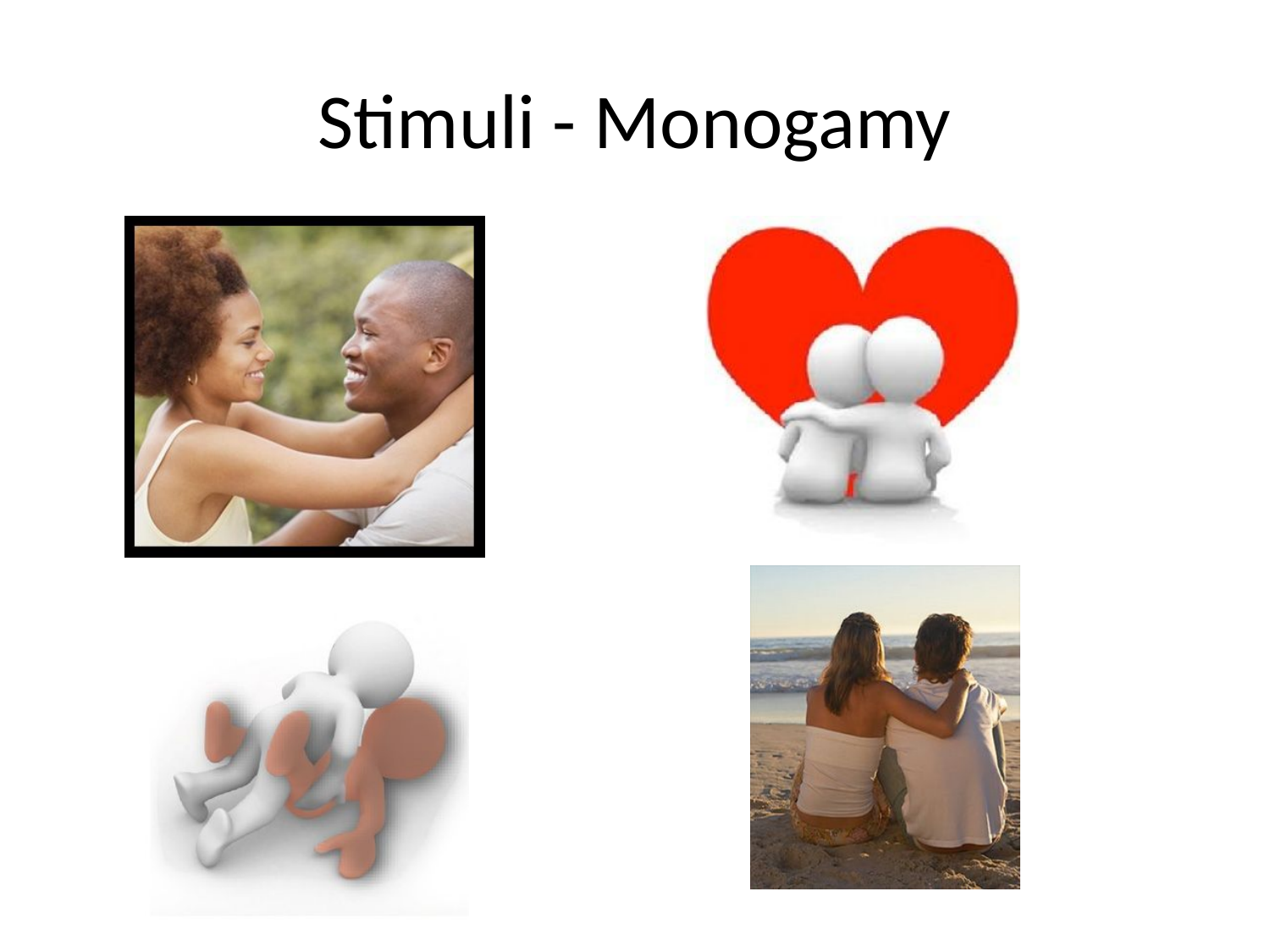

# Stimuli - Monogamy

## Slide 3
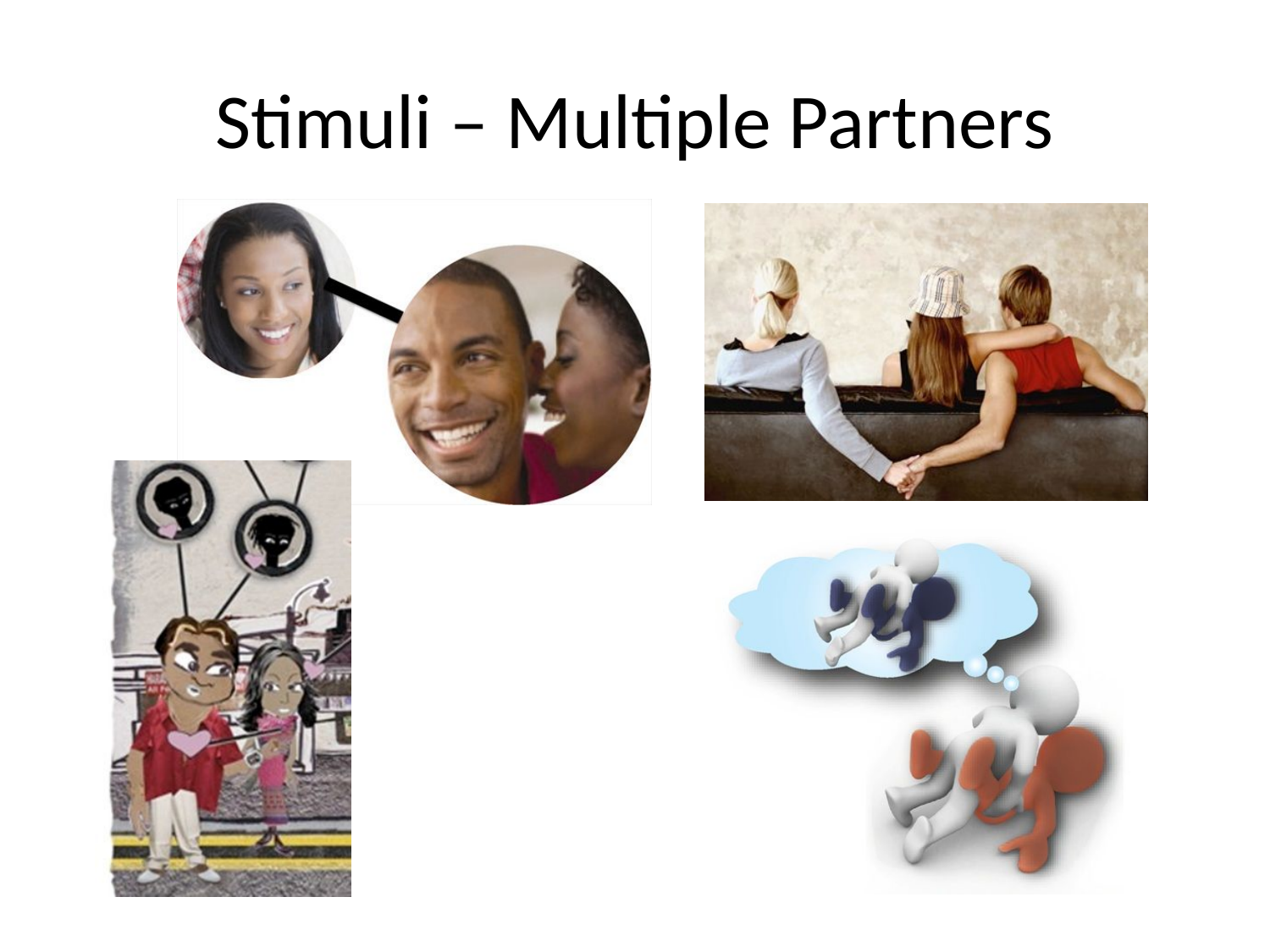

# Stimuli – Multiple Partners

## Slide 4
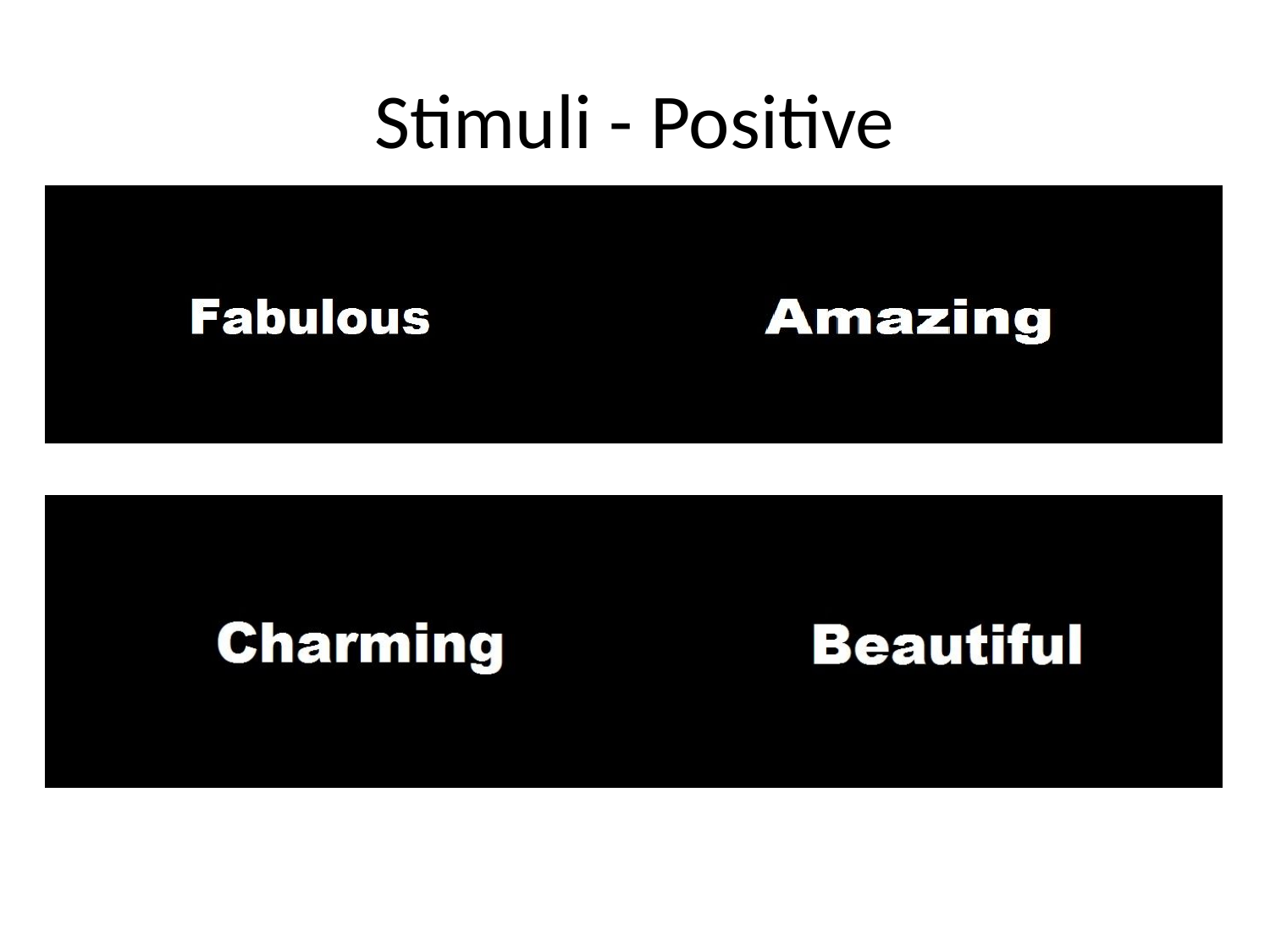

# Stimuli - Positive

## Slide 5
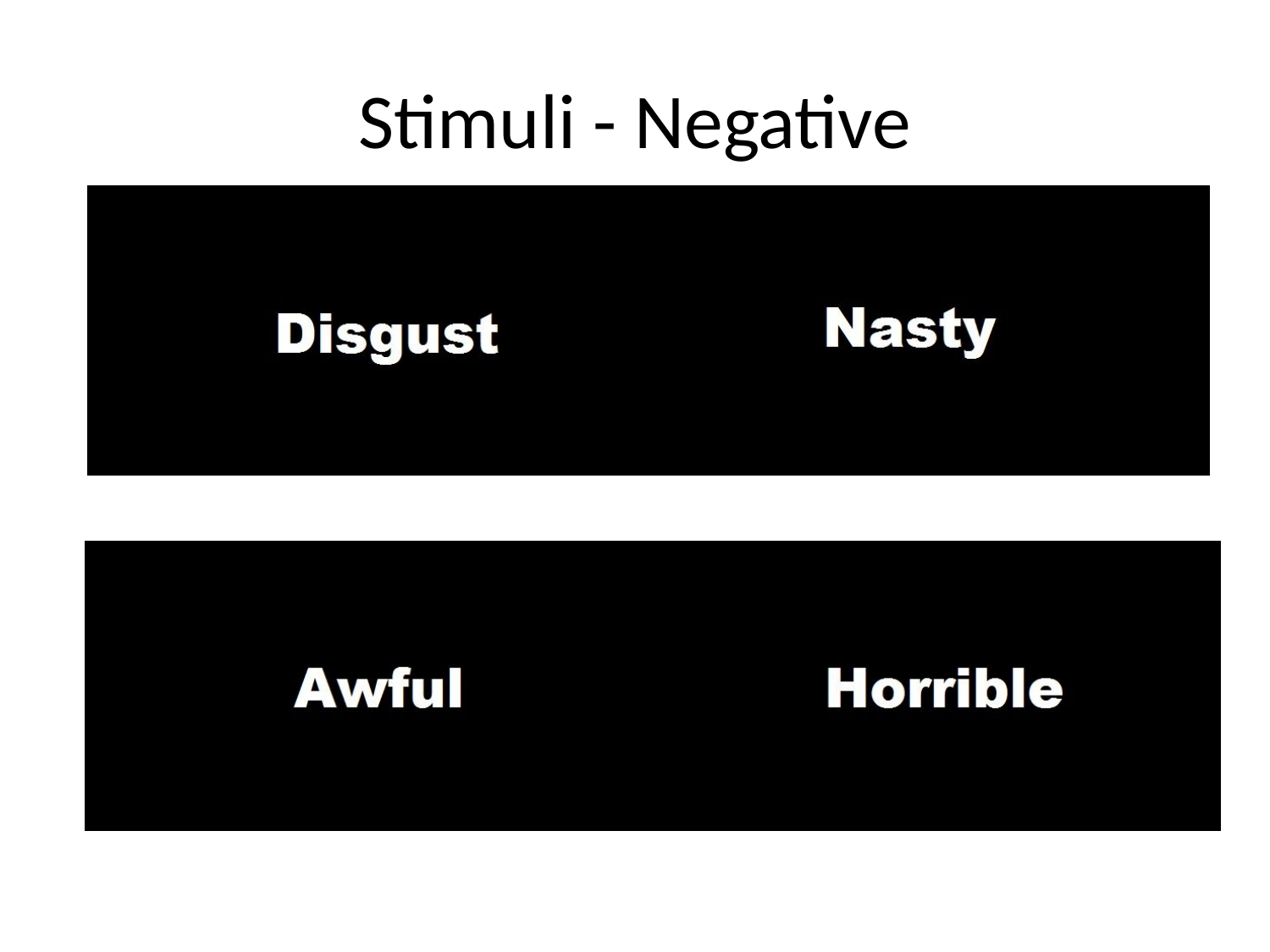

# Stimuli - Negative
