# Supplementary material for: Could differences in implicit attitudes to sexual concurrency play a role in generalized HIV epidemics?
Source: F1000Res. 2018 Oct 18;7:608. Originally published 2018 May 17. [Version 2] doi: 10.12688/f1000research.14951.2 (PMC6221060; doi:10.12688/f1000research.14951.2)
Supplement: Peer review history

# Peer review: Referee response for version 2

**DOI:** 10.5256/f1000research.18230.r39627

**Published:** 2018-11-06

**Authors:** Maddalena Marini

**Affiliations:** 1 Center for Translational Neurophysiology, Italian Institute of Technology (IIT), Ferrara, Italy

Abstract

 I see no changes in the conclusions of the abstract.

 Introduction

 Can authors discuss in the manuscript also the rationale and relevance of investigating the correlation between implicit and explicit attitudes at the individual and population levels as requested in my first report?

 Methods

 In the new online files I see no description of the IAT procedure but only stimuli used in the task. Can authors describe the IAT procedure in the text of the manuscript?

 Statistical analysis and results

 I suggest authors to provide the additional information about the computation of the D and explicit scores also in the text of the manuscript. In particular, since the sample size of the two groups was very different (869 vs. 70), how they calculated the Cohen’s d (and the associated pooled standard deviation).

I have read this submission. I believe that I have an appropriate level of expertise to confirm that it is of an acceptable scientific standard.

# Peer review: Referee response for version 2

**DOI:** 10.5256/f1000research.18230.r39626

**Published:** 2018-11-06

**Authors:** Hsun-Ta Hsu

**Affiliations:** 1 School of Social Work, University of Missouri, Columbia, MO, USA

I appreciate the authors addressing the comments I had; I do not have further comments.

I have read this submission. I believe that I have an appropriate level of expertise to confirm that it is of an acceptable scientific standard.

# Peer review: Referee response for version 1

**DOI:** 10.5256/f1000research.16276.r37802

**Published:** 2018-10-05

**Authors:** Maddalena Marini

**Affiliations:** 1 Center for Translational Neurophysiology, Italian Institute of Technology (IIT), Ferrara, Italy

The present study investigates the explicit and implicit attitudes to sexual concurrency in a sample of Belgium and South African students. Explicit attitudes were measured using self-reported items, while implicit attitudes were assessed by means of an Implicit Association Test (IAT). At the explicit level, both groups reported negative attitudes towards sexual concurrency, while at the implicit level, strong implicit preferences for monogamy were found only among Belgian students. No implicit preference between monogamy and multiple partners were indeed observed among South African students.

 General Comments:

 The aim of this study is very interesting because it highlights an implicit attitude to sexual concurrency that may underlie the origin of generalized HIV epidemic in South Africa. However, I have some reservations about the methodology, the statistical analysis, and the conclusions that prevent me from supporting the publication of this manuscript in its present form.

 Abstract

1. Authors state that their results “are compatible with the thesis that differences in implicit attitudes to concurrency play a role in the genesis of generalized HIV epidemics”. Since the present study did not investigate directly the relationship between implicit attitudes and HIV epidemic, I would suggest to be more careful in drawing these conclusions.

Introduction

1. On page 3, the authors state: “(ii) if the variation between these two populations involves a difference in behavior of ‘core-groups’ or general population shifts (iii) the correlation between implicit and explicit attitudes to concurrency and reporting that one has engaged in concurrency at both individual and population levels”. What did the authors exactly mean with ‘core-group’? Why is it important to evaluate the correlation between implicit and explicit attitudes at the individual and population levels? I suggest the authors to discuss more in details these points, their rationale and relevance.

Methods

1. For the IAT structure and its stimuli, authors refer to one of their previous publications ^1^. However, I would recommend the authors to provide a description of the IAT procedure as well as examples of stimuli used in their study, so that readers can better understand this instrument.

Statistical analysis

1. Could the authors please provide additional information on how they computed the D-scores?
2. In addition, it would great if the authors could also provide more details on the procedure used for individual and population level analyses.

Results

1. The authors state that “there was no difference between C-IAT results by gender in either country (data shown)”. I suggest the authors to report also the relative statistics.
2. Could the authors please provide additional information on how they computed the Cohen’s d scores reported in the results of implicit and explicit attitudes?
3. The r range of explicit measures reported in the text and in Table 2 is different. In the text, it is from 0.13 to 0.58, while in Table 2 it is from 0.08 to 0.58. Please clarify.

Discussions

1. Could the authors please clarify the following statement reported on page 6 “If this is the case and this acceptance is causally linked to higher concurrency rates then the distribution of implicit responses to concurrency amongst South African students suggests that population level interventions would be required to address this issue”?
2. The authors state “the C-IAT may constitute an important marker of a population-wide greater tolerance of concurrency which broadly enables higher concurrency rates but that other specific risk factors then determine which individuals will engage in concurrency”. Could they please elaborate on that?

Minor comment:

 I would suggest the authors to read carefully the text as it presents several typos and problems in the grammatical structure.

I have read this submission. I believe that I have an appropriate level of expertise to confirm that it is of an acceptable scientific standard, however I have significant reservations, as outlined above.

## References

[1] KenyonCWolfsKOsbakKvan LankveldJVan HalG: Implicit attitudes to sexual partner concurrency vary by sexual orientation but not by gender—A cross sectional study of Belgian students. *PLOS ONE*.2018;13(5) : 10.1371/journal.pone.0196821 10.1371/journal.pone.0196821 PMC594021329738541

# Response

**Published:** 2018-10-12

**Authors:** Chris Kenyon

**Affiliations:** Institute of Tropical Medicine, Antwerp, Belgium

General Comments:

The aim of this study is very interesting because it highlights an implicit attitude to sexual concurrency that may underlie the origin of generalized HIV epidemic in South Africa. However, I have some reservations about the methodology, the statistical analysis, and the conclusions that prevent me from supporting the publication of this manuscript in its present form.

Abstract

1. Authors state that their results “are compatible with the thesis that differences in implicit attitudes to concurrency play a role in the genesis of generalized HIV epidemics”. Since the present study did not investigate directly the relationship between implicit attitudes and HIV epidemic, I would suggest to be more careful in drawing these conclusions.

**Reply:**

**The abstract conclusion has been changed to the following:**

**South African students were found to have less of a preference for monogamy than Belgian students.**

Introduction

1. On page 3, the authors state: “(ii) if the variation between these two populations involves a difference in behavior of ‘core-groups’ or general population shifts (iii) the correlation between implicit and explicit attitudes to concurrency and reporting that one has engaged in concurrency at both individual and population levels”. What did the authors exactly mean with ‘core-group’? Why is it important to evaluate the correlation between implicit and explicit attitudes at the individual and population levels? I suggest the authors to discuss more in details these points, their rationale and relevance.

 

**Reply:**

**The following text has been added to the end of the introduction to make this clearer:**

**Our rationale for exploring if the variation between these two populations involves a difference in behavior of ‘core-groups’ or general population shifts is based on the work of Rose and others ^25^^– ^^27^. They argued that if one finds a bimodal distribution in behavior 'A' in population 'B' compared to a normal distribution in a comparison population 'C' then this finding would be compatible with the existence of a core-group with higher risk behaviour in population 'B' being responsible for some of the differences in behavior 'A' between the two groups. The concept of a 'core-group' is well established in the HIV field and typically refers to a subpopulation with a high level of sexual network connectivity (conferred by features such as partner concurrency and rate of partner change) that contributes disproportionately to the spread of HIV in that population . Kenyon CR, Tsoumanis A, Schwartz IS: A population's higher-risk sexual behaviour is associated with its average sexual behaviour-An ecological analysis of subpopulations in Ethiopia, Kenya, South Africa, Uganda and the United States. *Epidemics.* 2016;15:56–65. 27266849 10.1016/j.epidem.2016.02.002**

Methods

1. For the IAT structure and its stimuli, authors refer to one of their previous publications ^1^. However, I would recommend the authors to provide a description of the IAT procedure as well as examples of stimuli used in their study, so that readers can better understand this instrument.

**Reply:**

**This information has been provided as a two new online files:**

**S1 IAT Test. Concurrency implicit association test.**

**S2 Figures and words used in concurrency implicit association test.**

Statistical analysis

1. Could the authors please provide additional information on how they computed the D-scores?

**Reply:**

**The D600 scores were calculated as follows:**

**D600-scores of the IAT were calculated according to the standard protocol suggested by Greenwald et al. Reaction times of the second target-attribute combination were subtracted from the first combination, correcting for combination sequence, and divided by the pooled standard deviation of all practice and test phases. Scores usually vary between -2 and +2, with high scores indicating strong implicit**

**preferences for monogamy and concurrency, respectively, with zero indicating absence of preference, positive scores indicating a positive implicit association with concurrency (and a negative association with monogamy), and negative scores indicating a negative implicit association with concurrency (and a positive association with monogamy). Before calculating the D600 score, the minimum response time was set at 400 ms, the maximum response time at 2500ms. Any responses below this interval were omitted while any responses above this interval were recoded to 2500 ms. Reaction times of incorrect answers were raised using a penalty of**

**600 ms.**

1. In addition, it would great if the authors could also provide more details on the procedure used for individual and population level analyses.

**Reply:**

**For the population level analyses, Spearman’s correlation was used to assess the population level correlations between the point-prevalence of concurrency and intrinsic (mean D600) and extrinsic (mean values for each of the 3 variables considered separately) attitudes. The populations were defined according to self-reported sexuality by country and gender. Only populations with n > 10 were utilized for the analyses.**

**For the individual level analyses, the statistical analytical strategy used is described in the methods:**

**We compare the distributions of C-IAT (D600-scores) scores between Belgium and South Africa visually using histograms and statistically using t-tests for independent samples. In keeping with standard practice in this field, we used Cohen’s d as a measure of effect size. Cohen’s d was calculated by dividing the South African minus the Belgian mean difference D600 by the pooled standard deviation. Pearson’s correlation was used to test the correlations between implicit and explicit attitudes as well as between these two and self-reported point-prevalence of concurrency. Chi-squared and t-tests were used to test differences between categorical and continuous variables. All analyses were repeated stratified by gender. There were differences by gender in self-reported concurrency and explicit (but not implicit) attitudes towards concurrency. These differences were, however, congruent between Belgium and South Africa and did not affect the results. As a result, only unstratified results are reported.**

Results

1. The authors state that “there was no difference between C-IAT results by gender in either country (data shown)”. I suggest the authors to report also the relative statistics.

**Reply:**

**This information has been added to the second paragraph of the results as follows:**

**There was no difference in mean C-IAT score between men and women in Belgium (-0.81, SD = 0.39 and -0.78, SD = 0.40, respectively) or South Africa (0.05, SD = 0.46 and -0.01, SD = 0.40, respectively)**

1. Could the authors please provide additional information on how they computed the Cohen’s d scores reported in the results of implicit and explicit attitudes?

**Reply:**

**Cohen’s d was calculated by dividing the South African minus the Belgian mean difference D600 by the pooled standard deviation.**

1. The r range of explicit measures reported in the text and in Table 2 is different. In the text, it is from 0.13 to 0.58, while in Table 2 it is from 0.08 to 0.58. Please clarify.

**Reply:**

**Thank you for pointing this out. It has been corrected to 0.08 to 0.58 in the text.**

Discussions

1. Could the authors please clarify the following statement reported on page 6 “If this is the case and this acceptance is causally linked to higher concurrency rates then the distribution of implicit responses to concurrency amongst South African students suggests that population level interventions would be required to address this issue”?

**Reply:**

**We have rewritten this section to make it clearer. It now reads:**

**If our IAT results from South Africa are indeed reflective of a broader acceptance of concurrency than a population such as Belgium and this acceptance is causally linked to higher concurrency rates then the distribution of implicit responses to concurrency amongst South African students suggests that population level interventions would be required to address this issue.**

 

1. The authors state “the C-IAT may constitute an important marker of a population-wide greater tolerance of concurrency which broadly enables higher concurrency rates but that other specific risk factors then determine which individuals will engage in concurrency”. Could they please elaborate on that?

**Reply:**

**The following text has been added to this section to make it clearer:**

What this suggests is that there is a broader acceptance of concurrency in South Africa at an implicit level. This might play a role in determining the higher prevalence of concurrency in South Africa. Other factors such as previous experience of partner concurrency may then determine which specific individuals engage in concurrency .

Minor comment:

I would suggest the authors to read carefully the text as it presents several typos and problems in the grammatical structure.

**Reply:**

The paper has been re read and a number of typos corrected.

# Peer review: Referee response for version 1

**DOI:** 10.5256/f1000research.16276.r34132

**Published:** 2018-06-06

**Authors:** Hsun-Ta Hsu

**Affiliations:** 1 School of Social Work, University of Missouri, Columbia, MO, USA

**Introduction**

 1. I would recommend the authors do a final review of the paper to ensure it is free of grammatical errors and typos (e.g., in the first sentence in the introduction, "were" should be "where").

 2. The authors stated: "This discrepancy may be partly explained by the way that the social desirability bias may affect the accuracy of self-reported data pertaining to socially sensitive topics such as sexual norms." I would like to see more elaboration as it pertains to concurrency. 

 3. The statement regarding the findings of applying C-IAT on Belgium students is great. Since one of the study purposes is to identify the discrepancies between implicit and explicit norms between Belgium and South African participants, I would like to see the authors add their findings on whether there were discrepancies regarding explicit and implicit concurrency norms in their previous Belgium research. Currently, the authors only provide information regarding implicit norms of concurrency among Belgium students.

 4. It would be great if the authors would discuss their rationale for comparing South African and Belgium students.

 5. Also, it would be beneficial if the authors could provide more justification for the study aims (e.g., why look at shifts and correlations of the norms).

 6. Finally, the rationale of looking at college students should also be provided. Is it because they more likely to be sexually active? More likely to engage in concurrency? Or, more likely to at risks of HIV/STIs? 

**Method**

 1. Information regarding validity and reliability of the measurements used may be needed.

 2. The authors mentioned that they recruited participants who were "similar" to participants in the Belgium study for the comparison of implicit and explicit norms. How did the authors determine similarity when recruiting participants in South Africa? Since they are all college students, I am assuming their age might be similar? How about the concentrations/majors? 

 3. More info regarding sampling may be needed. For example, how many students were approached? What was the refusal rate? 

 4. The measurements were used to measure implicit and explicit norms. Why would there be incorrect answers? Could the authors provide some examples?

**Results**

 1. Some of the numbers stated in the text do not match the tables. For example, the author stated: "Self-reported concurrency behavior was slightly more strongly associated with explicit (r = 0.13 to 0.58) than implicit (r = 0.05 to 0.11) attitudes to concurrency by country." But, in Table 2, it seems that the correlation of explicit and concurrency behavior is 0.08-0.58. The authors might want to review the numbers.

**Discussion**

 1. The authors provide great information in that based on the finding, current efforts of targetting high-risk individuals may not be enough. Could the authors provide more information on, specifically, what may be the potential approach to address concurrency, especially targetting implicit attitudes on a population level?

 2. In the discussion section, the authors seemed to imply that the findings may apply to population level. However, given the sampling strategy, I am not sure if such argument may be the case. Could the authors provide more evidence or explanation?

I have read this submission. I believe that I have an appropriate level of expertise to confirm that it is of an acceptable scientific standard, however I have significant reservations, as outlined above.

# Response

**Published:** 2018-06-20

**Authors:** Chris Kenyon

**Affiliations:** Institute of Tropical Medicine, Antwerp, Belgium

Dear Dr Hsu

Thank you for your useful suggestions and comments, which we respond to below:

Introduction

1. I would recommend the authors do a final review of the paper to ensure it is free of grammatical errors and typos (e.g., in the first sentence in the introduction, "were" should be "where”).

Reply:

This will be done in the next version.

2. The authors stated: "This discrepancy may be partly explained by the way that the social desirability bias may affect the accuracy of self-reported data pertaining to socially sensitive topics such as sexual norms." I would like to see more elaboration as it pertains to concurrency. 

Reply:

This will be done in the next version of the paper.

3. The statement regarding the findings of applying C-IAT on Belgium students is great. Since one of the study purposes is to identify the discrepancies between implicit and explicit norms between Belgium and South African participants, I would like to see the authors add their findings on whether there were discrepancies regarding explicit and implicit concurrency norms in their previous Belgium research. Currently, the authors only provide information regarding implicit norms of concurrency among Belgium students.

Reply: 

These results are provided in detail at:

Kenyon CR, Wolfs K, Osbak K, et al.: Implicit attitudes to sexual partner

concurrency vary by sexual orientation but not by gender - a cross sectional

study of Belgian students. PLoS One. (In Press). 2018; 13(5): e0196821.

4. It would be great if the authors would discuss their rationale for comparing South African and Belgium students.

Reply:

This was based on opportunity sampling related to the institutions/countries where the authors work.

5. Also, it would be beneficial if the authors could provide more justification for the study aims (e.g., why look at shifts and correlations of the norms).

Reply:

This will be done in the next version of the paper.

6. Finally, the rationale of looking at college students should also be provided. Is it because they more likely to be sexually active? More likely to engage in concurrency? Or, more likely to at risks of HIV/STIs? 

Reply: 

Concurrency and STI rates are particularly high in this age group.

Method

1. Information regarding validity and reliability of the measurements used may be needed.

Reply:

As we note in this paper this was assessed in the previous paper we refer to:

Kenyon CR, Wolfs K, Osbak K, et al.: Implicit attitudes to sexual partner

concurrency vary by sexual orientation but not by gender - a cross sectional

study of Belgian students. PLoS One. (In Press). 2018; 13(5): e0196821.

2. The authors mentioned that they recruited participants who were "similar" to participants in the Belgium study for the comparison of implicit and explicit norms. How did the authors determine similarity when recruiting participants in South Africa? Since they are all college students, I am assuming their age might be similar? How about the concentrations/majors? 

Repy:

We did not collect information as to the subjects they were studying.

3. More info regarding sampling may be needed. For example, how many students were approached? What was the refusal rate? 

Reply: 

We do not have this data. The recruitment method used is detailed in the section:

In both countries all students at the two participating Universities

were eligible for study inclusion. In Belgium they were recruited

via an email sent to the entire student body. This was not possible

in South Africa and thus students were recruited via posters and

word of mouth.

4. The measurements were used to measure implicit and explicit norms. Why would there be incorrect answers? Could the authors provide some examples?

Reply:

The reference to “incorrect answers" on the IAT test refers to misclassifying an image or word during the IAT test e.g. placing an image of monogamy in the concurrency section.

Results

1. Some of the numbers stated in the text do not match the tables. For example, the author stated: "Self-reported concurrency behavior was slightly more strongly associated with explicit (r = 0.13 to 0.58) than implicit (r = 0.05 to 0.11) attitudes to concurrency by country." But, in Table 2, it seems that the correlation of explicit and concurrency behavior is 0.08-0.58. The authors might want to review the numbers.

Reply:

Thank you for pointing out this error which will be corrected in the next version of the paper.

Discussion

1. The authors provide great information in that based on the finding, current efforts of targetting high-risk individuals may not be enough. Could the authors provide more information on, specifically, what may be the potential approach to address concurrency, especially targetting implicit attitudes on a population level?

Reply:

This paragraph has now been expanded with the following text to address this question:

Current efforts targeting concurrency are largely focused on higher risk individuals which are unlikely to result in a shift in the population distribution in implicit attitudes to concurrency ^5, 27, 31 ^. One approach may be follow the Know Your Network concurrency reduction intervention which was shown to be feasible and acceptable in a rural Kenyan population ( **Knopf A, et al.** "This is the medicine:" A Kenyan community responds to a sexual concurrency reduction intervention. *Social Science & Medicine* 2014; **108**: 175-184).

2. In the discussion section, the authors seemed to imply that the findings may apply to population level. However, given the sampling strategy, I am not sure if such argument may be the case. Could the authors provide more evidence or explanation?

Reply:

This is true. In the new version we have changed this claim to the following weaker claim:

We found weak support for this latter interpretation in the form of a population level correlation between implicit attitudes towards concurrency and the practice thereof. This is however based on a small number of samples and this finding should be regarded as tentative.

# Response

**Published:** 2018-10-12

**Authors:** Chris Kenyon

**Affiliations:** Institute of Tropical Medicine, Antwerp, Belgium

**Thank you for your useful suggestions and comments, which we respond to below:**

Introduction

1. I would recommend the authors do a final review of the paper to ensure it is free of grammatical errors and typos (e.g., in the first sentence in the introduction, "were" should be "where”).

**Reply:**

**This has been done in the new version.**

2. The authors stated: "This discrepancy may be partly explained by the way that the social desirability bias may affect the accuracy of self-reported data pertaining to socially sensitive topics such as sexual norms." I would like to see more elaboration as it pertains to concurrency. 

**Reply:**

**The following text has been added to make this clearer:**

**Respondents to surveys asking about attitudes to sexual partner concurrency may consider that the interviewer holds negative attitudes towards concurrency. They may therefore bias their reported attitudes towards concurrency towards that of the interviewer.**

3. The statement regarding the findings of applying C-IAT on Belgium students is great. Since one of the study purposes is to identify the discrepancies between implicit and explicit norms between Belgium and South African participants, I would like to see the authors add their findings on whether there were discrepancies regarding explicit and implicit concurrency norms in their previous Belgium research. Currently, the authors only provide information regarding implicit norms of concurrency among Belgium students.

**Reply:**

**These results are provided in detail at:**

**Kenyon CR, Wolfs K, Osbak K, et al.: Implicit attitudes to sexual partner**

**concurrency vary by sexual orientation but not by gender - a cross sectional**

**study of Belgian students. PLoS One. 2018; 13(5): e0196821.**

4. It would be great if the authors would discuss their rationale for comparing South African and Belgium students.

**Reply:**

**This was based on opportunity sampling related to the institutions/countries where the authors work. Various studies have noted concurrency prevalences to be relatively low in Western Europe and relatively high in South Africa** **^1-3^** **.**

5. Also, it would be beneficial if the authors could provide more justification for the study aims (e.g., why look at shifts and correlations of the norms).

**Reply:**

**The following text has been added to the end of the introduction to make this clearer:**

**Our rationale for exploring if the variation between these two populations involves a difference in behavior of ‘core-groups’ or general population shifts is based on the work of Rose and others** **^25^** **^–^** **^27^** **. They argued that if one finds a bimodal distribution in behavior 'A' in population 'B' compared to a normal distribution in a comparison population 'C' then this finding would be compatible with the existence of a core-group with higher risk behaviour in population 'B' being responsible for some of the differences in behavior 'A' between the two groups. The concept of a 'core-group' is well established in the HIV field and typically refers to a subpopulation with a high level of sexual network connectivity (conferred by features such as partner concurrency and rate of partner change) that contributes disproportionately to the spread of HIV in that population .** **Kenyon CR, Tsoumanis A, Schwartz IS: A population's higher-risk sexual behaviour is associated with its average sexual behaviour-An ecological analysis of subpopulations in Ethiopia, Kenya, South Africa, Uganda and the United States. *Epidemics.* 2016;15:56–65. 27266849 10.1016/j.epidem.2016.02.002**

**In addition, we have explained the rationale for looking at shifts in C-IAT between Belgium and South Africa in the following text in the discussion:**

**The IAT results for the study populations in South Africa and Belgium were both normally distributed with a similar variance. Belgium’s population curve was however relatively right-shifted. There was no evidence of a ‘core high risk group’ with a distribution outside of the Gaussian distribution in either country. This variation of distributions between different populations via right or left shifting the mean value (but retaining the same variance) mimics the findings of Rose and others for a wide variety of physical and mental health attributes and behaviors, including number of sex partners ^25^^– ^^27^ . Rose’s interpretation of this relationship was that populations do not tolerate ‘excessive’ variations in norms and behaviors and thus distributions of these characteristics move up and down as a whole ^27^ .**

**As far as assessing the correlation between C-IAT and explicit norms is concerned, this is a standard assessment in this type of IAT research. We have provided this rationale in the following text in the discussion:**

**A limitation with the line of reasoning outlined above is the low correlation found between self-reported concurrency and implicit attitudes to concurrency. This may be because the implicit attitudes play little or no role in driving high concurrency rates. Alternatively, the C-IAT may constitute an important marker of a population-wide greater tolerance of concurrency which broadly enables higher concurrency rates but that other specific risk factors then determine which individuals will engage in concurrency ^32^ . We found some support for this latter interpretation in the form of a population level correlation between implicit attitudes towards concurrency and the practice thereof.**

6. Finally, the rationale of looking at college students should also be provided. Is it because they more likely to be sexually active? More likely to engage in concurrency? Or, more likely to at risks of HIV/STIs? 

**Reply:**

**Concurrency and STI rates are particularly high in this age group** **^3, 4^** **.**

Method

1. Information regarding validity and reliability of the measurements used may be needed.

**Reply:**

**As we note in the current paper the assessment of validity and reliability were perfomed in the previous paper reporting the development of the C-IAT:**

**Kenyon CR, Wolfs K, Osbak K, et al.: Implicit attitudes to sexual partner**

**concurrency vary by sexual orientation but not by gender - a cross sectional**

**study of Belgian students. PLoS One. 2018; 13(5): e0196821.**

2. The authors mentioned that they recruited participants who were "similar" to participants in the Belgium study for the comparison of implicit and explicit norms. How did the authors determine similarity when recruiting participants in South Africa? Since they are all college students, I am assuming their age might be similar? How about the concentrations/majors? 

**Repy:**

**We did not collect information as to the subjects they were studying.**

3. More info regarding sampling may be needed. For example, how many students were approached? What was the refusal rate? 

**Reply:**

**We do not have this data. The recruitment method used is detailed in the following section:**

**In both countries all students at the two participating Universities**

**were eligible for study inclusion. In Belgium they were recruited**

**via an email sent to the entire student body. This was not possible**

**in South Africa and thus students were recruited via posters and**

**word of mouth.**

4. The measurements were used to measure implicit and explicit norms. Why would there be incorrect answers? Could the authors provide some examples?

**Reply:**

**The reference to “incorrect answers" on the IAT test refers to misclassifying an image or word during the IAT test e.g. placing an image of monogamy in the concurrency section. This is standard terminology in the IAT field.**

Results

1. Some of the numbers stated in the text do not match the tables. For example, the author stated: "Self-reported concurrency behavior was slightly more strongly associated with explicit (r = 0.13 to 0.58) than implicit (r = 0.05 to 0.11) attitudes to concurrency by country." But, in Table 2, it seems that the correlation of explicit and concurrency behavior is 0.08-0.58. The authors might want to review the numbers.

**Reply:**

**Thank you for pointing out this error which has been corrected in the new version of the paper.**

Discussion

1. The authors provide great information in that based on the finding, current efforts of targetting high-risk individuals may not be enough. Could the authors provide more information on, specifically, what may be the potential approach to address concurrency, especially targetting implicit attitudes on a population level?

**Reply:**

**This paragraph has now been expanded with the following text to address this question:**

**Current efforts targeting concurrency are largely focused on higher risk individuals which are unlikely to result in a shift in the population distribution in implicit attitudes to concurrency ^5, 27, 31 ^. One approach may be follow the Know Your Network concurrency reduction intervention which was shown to be feasible and acceptable in a rural Kenyan population ( Knopf A, et al. "This is the medicine:" A Kenyan community responds to a sexual concurrency reduction intervention. *Social Science & Medicine* 2014; 108: 175-184).**

2. In the discussion section, the authors seemed to imply that the findings may apply to population level. However, given the sampling strategy, I am not sure if such argument may be the case. Could the authors provide more evidence or explanation?

**Reply:**

**This is true. In the new version we have changed this claim to the following weaker claim:**

**We found weak support for this latter interpretation in the form of a population level correlation between implicit attitudes towards concurrency and the practice thereof. This is however based on a small number of samples and this finding should be regarded as tentative.**

**References**

1.         Leridon H, van Zessen G, Hubert M. The Europeans and their sexual partners. *Sexual behaviour and HIV/AIDS in Europe: Comparisons of national surveys *1998: 165-96.

2.         Kenyon C, Buyze J, Colebunders R. HIV prevalence by race co-varies closely with concurrency and number of sex partners in South Africa. *PLoS One *2013; **8**: e64080.

3.         Mah T. Prevalence and correlates of concurrent sexual partnerships among young people in South Africa. *Sex Transm Dis *2010; **37**: 105-8.

4.         Maughan-Brown B, Kenyon C, Lurie MN. Partner Age Differences and Concurrency in South Africa: Implications for HIV-Infection Risk Among Young Women. *AIDS and Behavior *2014: 1-8.
